# Philadelphia Hospital

**Published:** 1843-02-04

**Authors:** J. P. Tabb

**Affiliations:** Resident Surgeon


### PHILADELPHIA HOSPITAL.

### PROFESSOR PANCOAST'S CLINIC.

*January* 14, 1843.

[Reported by J. P. Tabb, Resident Surgeon.]

(Concluded.)

CASE IV.—The lecturer next exhibited a man with chronic enlargement of the tonsil gland of each side; and remarked, that in this case, though from the size of the tumours there was much inconvenience felt in deglutition, with stertorous breathing in his sleep, and sense of strangling, that occasionally waked him up; still it was not from either of these causes that the patient was desirous to have them removed, so much as the constant tendency of these swollen and spongy tumours to give rise to an attack of quinsy, on every exposure to cold. In the forming state of such tumours, and especially in scrofulous children, he had found astringent applications, and touching with lunar caustic, to cause them to disappear; but in general, when fully formed, he preferred excision as being more speedy, certain, and not dangerous. In former times surgeons were in the habit of removing them by a canula and wire ligature, strangulating the tumours at their base, which afterwards sloughed off. This plan was painful, tedious, uncleanly, and even dangerous.

The European surgeons, in excision of these parts, usually employ a hook or toothed forceps to seize the part, and remove it with the scissors or bistoury. The only instruments really well suited to this operation are of American invention. The ones which the lecturer commonly used were the guillotine instrument of Physick, as modified by Dr. J. K. Mitchell; the ring instrument of Fahnestock, with a knife nearly circular in shape; and a modification of this, with a pair of forceps and spring upon the front, which of itself draws out the tumour from between the half arches, so as to insure the removal of a sufficiently large portion. To the latter instrument he was inclined to give the preference, as it required but the use of one hand. A tonsil was then removed without difficulty by each of the two latter instruments; which, as the edge of the knife could not from its shape be made quite so keen as the chisel of the guillotine instrument, he thought was less likely to be followed by troublesome bleeding. No instrument is usually necessary to hold down the tongue. All the precaution necessary in the operation is to accustom the fauces to the contact of instruments, by frequently touching them for several days previous with the handle of a spoon; and to be careful not to incise the half arches, which, as shown by Dr. I. Parrish, might be followed by some difficulty in enunciation.

Case V—was a fracture of the clavicle just beyond the middle, presenting the usual deformity of the shoulder following the injury. The accident was of 11 days standing. Dessault's bandage had been applied, which had not answered well, the fault being rather with the patient, who was exceedingly restless. The apparatus of Dr. Fox was shown and applied, which Dr. P. preferred in the treatment of this injury to Dessault's, as fulfilling the indications of treatment equally well, being less liable to derangement, and leaving the patient more at ease. The injury had occurred by a fall from a second story window, upon the elbow and trochanter major of the left side; the whole region of the hip having sustained a violent contusion, as shown by the extensive effusion of blood below the skin. He took occasion to call the attention of the class to the influence of the age of patients in modifying such accidents about the hip. For a fracture of the neck of the thigh-bone would most likely have been produced in addition, if the patient had been advanced in life. He had found this result often produced in this way, and occasionally seen the fragment of bone so interlocked by the force of the blow, as to maintain a straight position of the foot for several days after the accident. In the present case, though there was neither fracture nor displacement, some indistinct crepitation was heard on ratation of the limb, perceptible to the patient, and which he called slipping of the bone out of its place; but which was attributable merely to the friction of the trochanter against the thickened and inflamed cellular tissue of the part.

Case VI.—Was a severe case of frost-bite of both hands, which having occurred at sea, had been improperly treated. The specific character of the affection was now lost; the sloughs were beginning to separate, and required nothing more than the ordinary treatment for chronic ulcers, poultices, till the sloughs were discharged, and slightly stimulating applications afterwards, attention being given to keep the fingers separate, and prevent adhesions between them.

Case VII.—*Operation for Hydrocele.*—This wasan accumulation of fluid in the tunica vaginalis testis, that had accompanied a chronic enlargement of the testicle, and remained after the latter had been completely discussed by treatment. The case having before come under the notice of the class, and the lecturer having on the occasion of a former operation entered fully into the nature of hydrocele and its treatment, he at once injected the sac with a fluid consisting of one part of Tr. of Iodine and three of water; a measure of proceeding which had been invariably successful in his hands.

Case VIII.—*Amputation of the toe, at the metatarso-phalangeal joint.* This was a case of dislocation of the small toes of long standing, resulting from a fall of some bricks from a height on the dorsum of the foot. The points of the toes were directed outwards to the margin of the foot. The small toe had been removed by the lecturer a year ago, in consequence of its preventing the wearing of a shoe, by its projection beyond the margin of the foot. Attention was called to the covering of the stump, which was soft and moveable, a result which is obtained only when we operate upon either margin of the foot, by giving the greatest length possible to the outer flap, as there is a disposition to the retraction of the marginal integuments, and the exposure of the end of the metatarsal bone. A similar degree of inconvenience as the first is now experienced from the second small toe. This was removed by the oval operation, commencing it at the top of the joint and circumscribing in the first incision the commissure of the toes. To this operation the lecturer gave a decided preference over that of Lisfranc, which divides the thickened and sensitive covering of the sole of the foot, and is apt to leave a painful cicatrix. No dressing was required but a single strip of adhesive plaster to close the V shaped wound that was left on the removal of the toe.

